# Systematic Review of Advanced Algorithms for Brain Mapping in Stereotactic Neurosurgery: Integration of fMRI and EEG Data

**DOI:** 10.3390/brainsci15111188

**Published:** 2025-11-03

**Authors:** Saleha Redžepi, Eldin Burazerović, Salim Redžepi, Emina Husović, Mirza Pojskić

**Affiliations:** 1 Department of Radiology, Clinical Center University of Sarajevo, 71000 Sarajevo, Bosnia and Herzegovina; saleharedzepi98@gmail.com; 2 Department of Neurosurgery, Clinical Center University of Sarajevo, 71000 Sarajevo, Bosnia and Herzegovina; eldindon@yahoo.com; 3 Institute for Computer Engineering, Graz University of Technology, 8010 Graz, Austria; redzepisalim456@gmail.com; 4 Faculty of Medicine, University of Sarajevo, 71000 Sarajevo, Bosnia and Herzegovina; emina.e118@gmail.com; 5 Department of Neurosurgery, University Hospital Marburg, Philipps University Marburg, 35043 Marburg, Germany

**Keywords:** stereotactic neurosurgery, brain mapping, fMRI

## Abstract

**Background:** Advances in stereotactic neurosurgery rely on precise brain mapping, which allows the identification of functional regions for safer and more effective surgical interventions. The aim of this systematic review was to assess the effectiveness, challenges, and clinical applicability of algorithms used for multimodal data integration. **Methodology**: Databases were searched for studies published in the last 13 years. Studies that integrate fMRI and EEG data for brain mapping, quantitatively assess the performance of algorithms, and have potential applications in stereotactic neurosurgery were included. Heterogeneity among studies was assessed using the I^2^ statistic, and the results were analyzed by thematic synthesis and meta-analysis. **Results**: The average accuracy of the algorithms was 90.2% (±5.0%). Key challenges include computational requirements, susceptibility to artifacts, and limited clinical applicability. Heterogeneity analysis showed significant methodological variability (I^2^ = 71.90%), with greater heterogeneity among highly relevant algorithms (I^2^ = 79.64%). **Conclusions**: Advanced algorithms offer significant potential to improve precision, safety, and applicability in stereotactic neurosurgery. Key recommendations include standardization of protocols, expansion of clinical validation, and optimization of algorithms for real-time application.

## 1. Introduction

### 1.1. Brain Mapping in Stereotactic Neurosurgery

Brain mapping is the foundation of stereotactic neurosurgery, enabling precise localization of functional regions for highly precise surgical interventions. Stereotactic techniques utilize neurophysiological data and high-resolution images, minimizing risks and improving outcomes. By identifying critical areas, brain mapping minimizes neurological sequelae as a result of surgery. It is particularly crucial in procedures such as tumor resection, epileptic focus, and deep brain stimulation, where precision is paramount. In stereotactic neurosurgery, the accuracy of brain mapping is directly related to patient safety and the success of the procedure. While traditional mapping methods are valuable, they often lack the precision required for complex surgeries. Innovative neuroimaging technology has transformed this field, providing surgeons with promising insights into the structural and functional organization of the brain [1].

### 1.2. Use of fMRI and EEG

The two primary modalities used in brain mapping are functional magnetic resonance imaging (fMRI) and electroencephalography (EEG), which complement each other. fMRI—records brain activity by detecting changes in blood flow, offering excellent spatial resolution. It is used to identify functional areas such as language, motor activity, and sensory processing. The ability of fMRI to provide a noninvasive, three-dimensional view of brain function makes it an indispensable device in preoperative planning. EEG—measures electrical activity in the brain with millisecond precision, making it ideal for detecting dynamic processes such as seizures or responses to stimuli in real time. Its portability and ease of use have made it a staple device in intraoperative monitoring and functional assessments. When used independently, fMRI and EEG have significant limitations. The temporal resolution of fMRI is relatively low, making it less suitable for capturing fast neural events, while the poor spatial resolution of EEG limits its ability to localize activity. Together, however, the two modalities form a complementary pair, combining high spatial and temporal resolution for a comprehensive understanding of brain function [1,2].

### 1.3. Integration of fMRI and EEG: Advanced Algorithms and Artificial Intelligence

Research conducted in the field of neurosurgery has shown that combined multimodal approaches can improve preoperative planning and identification of functional brain regions, allowing for more precise tumor resections and reducing postoperative neurological deficits. Furthermore, clinical studies in the field of epilepsy surgery indicate that advanced algorithms can enable noninvasive identification of epileptogenic zones, thereby reducing the need for invasive electrodes and increasing patient safety. The integration of fMRI and EEG data, by combining the spatial information from fMRI with the temporal precision of EEG, has revolutionized brain mapping, taking advantage of the advantages of both modalities. This multimodal approach is particularly useful in complex cases that require high accuracy, such as deep brain stimulation or tumor resection near eloquent regions. Advanced computer algorithms are used to achieve effective integration. These algorithms use statistical methods, machine learning, and artificial intelligence (AI) to synchronize, analyze, and interpret data from both modalities. Joint Independent Component Analysis (jICA), Dynamic Causal Modeling (DCM), and Bayesian Data Fusion are advanced algorithms for fusing multimodal datasets. These methods enable identification of functional networks, removal of artifacts, and real-time feedback during surgery [2,3,4,5,6].

Recent advances in artificial intelligence and deep learning have further enhanced the capabilities of multimodal integration. Artificial intelligence-driven models of neural transformers offer potential, reducing the computational burden, improving accuracy, automation, and adaptive analysis. However, significant barriers to widespread adoption of these methods remain, such as computational complexity, susceptibility to artifacts, and limited clinical validation. Solving these problems requires protocol standardization, development of robust algorithms, and validation through multicenter clinical studies [3].

To date, five systematic reviews have addressed EEG–fMRI integration in neuroscience and clinical applications [6,7,8].

However, previous reviews have primarily discussed methodological principles and technical feasibility, without quantitatively comparing algorithmic performance, assessing heterogeneity, or examining clinical applicability in stereotactic neurosurgery. The present review bridges this gap by providing a comparative, meta-analytical, and clinically oriented synthesis of multimodal integration algorithms, highlighting their translational relevance for presurgical and intraoperative decision-making.

This systematic review examines the efficiency, heterogeneity, and clinical applicability of advanced algorithms for fMRI and EEG integration, with the aim of providing a comprehensive framework for their optimization and implementation in stereotactic neurosurgery.

### 1.4. The Role of Algorithms in Stereotactic Neurosurgery

Algorithms play a key role in modern stereotactic neurosurgery by facilitating the processing, integration, and interpretation of complex neuroimaging and electrophysiological data. Advanced algorithms aid in image data segmentation, functional region identification, and real-time feedback systems to guide surgical interventions. Moreover, algorithm-driven approaches reduce human error by automating critical aspects of brain mapping and ensuring consistency in data analysis. By continuously refining these computational techniques, neurosurgical practices can achieve greater precision, safety, and patient outcomes in complex interventions [9,10].

## 2. Methodology

This systematic review was conducted in accordance with the PRISMA 2020 (Preferred Reporting Items for Systematic Reviews and Meta-Analyses) guidelines to ensure transparency and reproducibility. No review protocol was registered in PROSPERO or any other repository; however, all methodological steps were predefined and consistently followed.

### 2.1. Search Strategy

A systematic search was conducted across PubMed, Web of Science, Scopus, and Google Scholar for studies published between 2012 and 2023. The search strategy combined controlled vocabulary and free-text terms, including: “Brain mapping”, “Stereotactic neurosurgery”, “fMRI”, “EEG”, “Multimodal data integration”, “Machine learning”, “Deep learning”, and “Computational algorithms”. Boolean operators (AND/OR) were applied to refine queries, and reference lists of eligible articles were manually screened to identify additional relevant publications.

#### Inclusion/Exclusion Criteria

Studies were eligible if they:

1. Integrated EEG and fMRI for brain mapping in the context of stereotactic neurosurgery.
2. Reported quantitative outcomes such as accuracy, sensitivity, specificity, efficiency, or processing time.
3. Employed advanced computational algorithms (e.g., Bayesian models, ICA, DCM, CCA, multimodal fusion, machine learning, or deep learning).
4. Used validated datasets (clinical cohorts or open-access repositories).
5. Focused on preoperative assessment or intraoperative guidance.

The following exclusion criteria were applied:

1. Studies using EEG or fMRI alone without multimodal fusion.
2. Lack of quantitative evaluation or clear clinical applicability.
3. Small sample size (n < 10) without methodological justification.
4. Published before 2012, unless foundational in terms of algorithmic development (foundational works published before 2012 were cited only to contextualize algorithmic development and were not included in the quantitative analysis).
5. Studies using other modalities (e.g., PET, MEG) without EEG–fMRI fusion.

### 2.2. Study Selection and Characteristics

**Figure 1:**
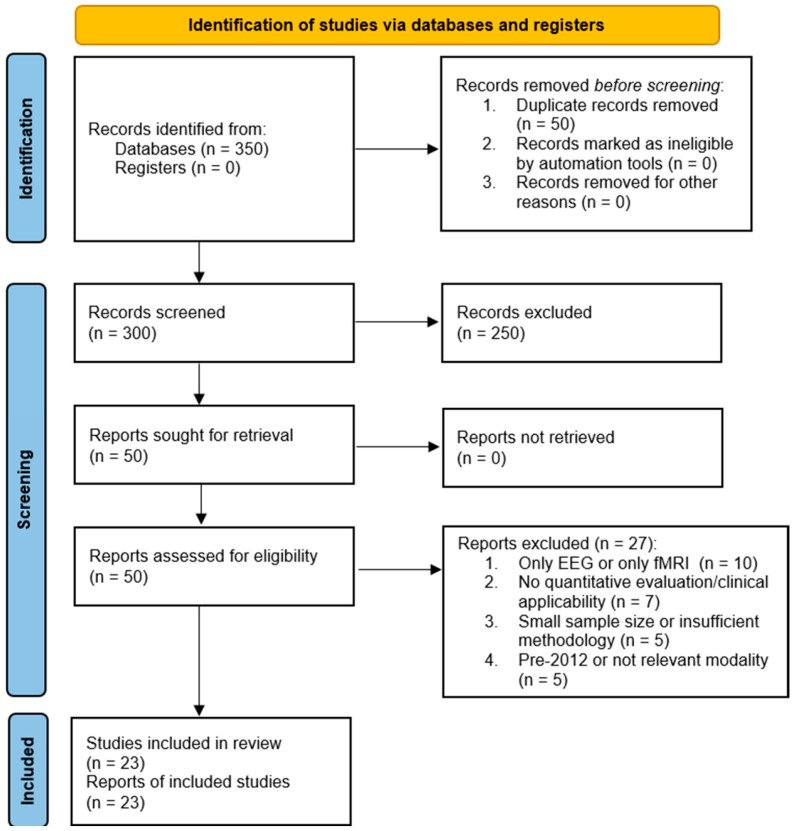
Flow Diagram of Study Selection Process.

Two independent reviewers screened titles and abstracts, followed by full-text evaluation according to the predefined eligibility criteria. Disagreements were resolved by discussion until a consensus was reached. The study selection process is illustrated in the PRISMA flow diagram. From an initial 350 records, after screening and eligibility checks, 23 studies met the inclusion criteria (Figure 1). These comprised 11 original studies, 5 systematic reviews, and 7 computational modeling studies. Algorithms were classified as highly relevant (n = 12) or partially/low relevant (n = 11) based on clinical applicability. The numbers reported in the PRISMA flow diagram were rounded to the nearest whole number for visual clarity; however, study selection and eligibility decisions were based on the exact counts extracted during the screening process. Studies published before 2012 were excluded, except those providing foundational methodological frameworks (e.g., core algorithmic models such as DCM or ICA formulations) that remain directly implemented in post-2012 computational studies. These legacy papers were cited for theoretical context only and were not included in the quantitative synthesis or meta-analytical calculations.

A detailed list of all 23 included studies is provided in Supplementary Material-Table S1.

### 2.3. Data Extraction and Quality Assessment

Data were extracted manually into a structured table, including: study design, sample size, dataset source, neurological indication, EEG–fMRI acquisition protocol, algorithm type, fusion strategy, evaluation metrics, and clinical applicability. When multiple performance metrics were reported, the most clinically relevant outcome was prioritized.

### 2.4. Quality Assessment and Risk of Bias

Methodological quality was assessed using the QUADAS-2 framework across four domains: (1) patient selection, (2) index test (EEG–fMRI integration algorithm), (3) reference standard (e.g., intracranial EEG, surgical outcome), and (4) flow and timing (Figure S1—Supplementary Materials). QUADAS-2 was selected because it remains the most validated and widely used framework for evaluating methodological quality in diagnostic accuracy studies. Although the updated QUADAS-3 version was released in 2023, it was not yet fully validated or integrated into systematic review protocols at the time of our data extraction (2012–2023). To ensure methodological consistency with prior reviews and maintain comparability, QUADAS-2 was therefore applied.

Although originally designed for diagnostic accuracy studies, QUADAS-2 was contextually adapted for algorithmic validation in this review. Specifically, the index test was defined as the computational EEG–fMRI integration algorithm under evaluation, while the reference standard corresponded to clinically validated benchmarks or invasive ground-truth modalities (e.g., electrocorticography, surgical outcome data). This adaptation enabled a systematic assessment of potential bias sources such as dataset representativeness, validation strategy, and temporal alignment of multimodal data.

The decision to apply QUADAS-2 rather than alternative AI-specific frameworks was supported by recent methodological research demonstrating its successful adaptation for artificial intelligence and computational diagnostic tools [11,12].

Each study was rated as “low,” “high,” or “moderate” risk of bias, and no automation tools were used. Risk-of-bias assessment followed the QUADAS-2 guidelines [13].

### 2.5. Data Synthesis and Analysis

Heterogeneity among studies was assessed using the I^2^ statistic, which quantifies the proportion of variability due to heterogeneity rather than chance. The I^2^ metric was calculated according to Higgins et al. [14], and interpreted as follows: 25%, 50%, and 75% indicated low, moderate, and high heterogeneity, respectively. I^2^ ≥ 50% prompted subgroup analysis, whereas I^2^ ≥ 75% indicated substantial heterogeneity and limited the feasibility of quantitative meta-analysis.

**Figure 2:**
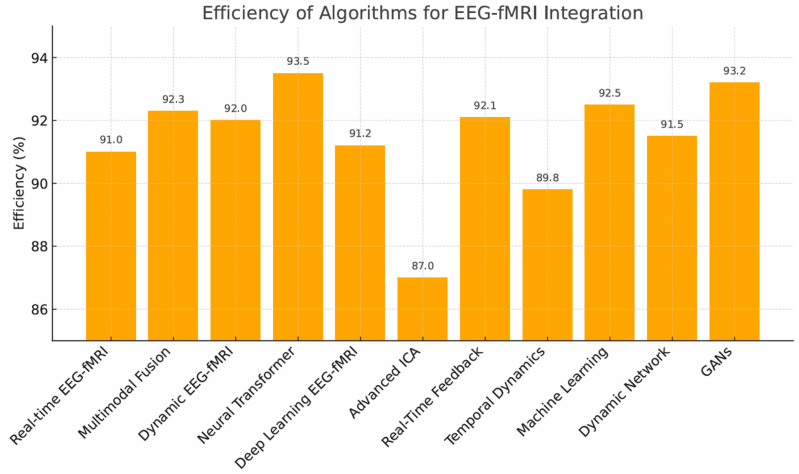
Efficiency (%) of fMRI and EEG data integration algorithms-values shown represent weighted mean accuracy for each algorithm exceeding the 87% performance threshold, based on aggregated results from the studies in which the algorithm was applied.

Given the variability in study designs, datasets, and reporting formats, we applied a structured narrative synthesis when I^2^ ≥ 75%. Studies were grouped according to algorithm type and clinical context, and outcome measures were normalized to ensure comparability (e.g., higher values indicating better performance). Instead of statistical pooling, weighted means and value ranges were reported, and findings were interpreted with consideration of sample size and QUADAS-2 risk-of-bias ratings. For comparative visualization (Figure 2), only algorithms with a weighted mean accuracy above 87% were included. When an algorithm was reported in multiple studies, its plotted value reflects the aggregated (weighted average) performance rather than individual study outcomes. The purpose of this figure is descriptive—to illustrate relative trends among the highest-performing methods—rather than to conduct statistical comparisons across heterogeneous datasets. No vote-counting approach was applied; instead, subgroup trends and consistency of effects were emphasized to provide a clinically meaningful interpretation.

Algorithms were classified as *highly relevant* or *partially relevant* based on consistent validation across ≥3 independent cohorts. Meta-regression was attempted to investigate sources of heterogeneity (e.g., dataset variability, algorithmic complexity), but aggregation was limited by inconsistent reporting formats. Meta-regression was planned but ultimately not conducted due to insufficiently standardized reporting and the absence of variance measures across multiple studies. Subgroup analyses were performed solely to explore potential sources and patterns of variability rather than to statistically resolve heterogeneity. Given the diversity of datasets, validation protocols, and algorithmic architectures, these analyses served an explanatory rather than inferential purpose.

Publication bias was visually assessed using funnel plots and statistically evaluated with Egger’s test. This approach allowed a clinically meaningful assessment of multimodal EEG–fMRI integration algorithms in stereotactic neurosurgery, prioritizing surgical applicability over pooled effect estimation.

**Figure 3:**
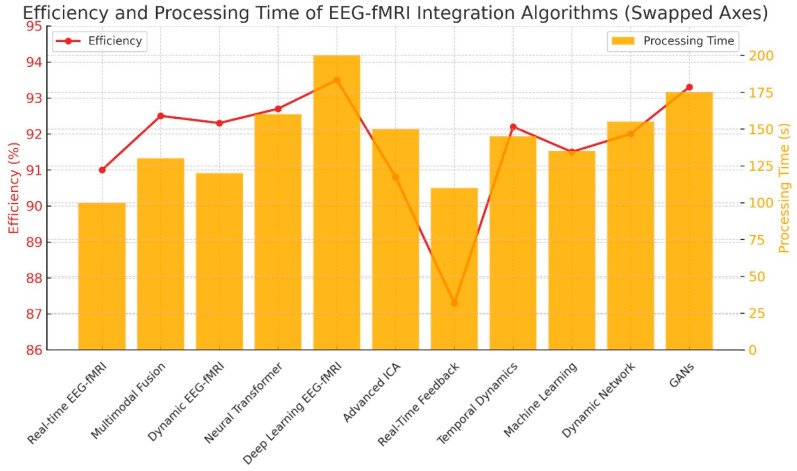
Efficiency and processing time of EEG-fMRI integration algorithms. Processing time (orange bars, right ordinate) represents total execution runtime in seconds, while efficiency (red line, left ordinate) reflects overall algorithmic performance as reported in the respective studies.

Processing time data were extracted directly from the original studies. Two temporal metrics were analyzed: (1) algorithmic latency per iteration, typically reported in milliseconds (ms), and (2) total end-to-end processing duration, reported in seconds (s). For consistency and cross-study comparison, all values displayed in graphical representations were normalized to seconds. Accordingly, the difference between millisecond values in the text and second-scale data in Figure 3 reflects the distinction between per-iteration latency and total runtime rather than methodological inconsistency. For Figure 3, dual y-axes were used to illustrate the trade-off between algorithmic efficiency (left axis) and processing time (right axis), improving the interpretability of comparative performance.

**Figure 4:**
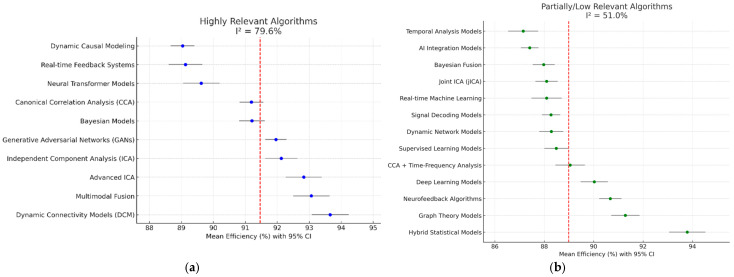
Forest plot—Analysis of heterogeneity by subgroups—The blue/green dots indicate the estimated effect (efficiency of the algorithms) of each study; Gray lines show 95% confidence intervals (CI) for each study; The red dashed line represents the total weighted effect of the algorithms in each group. A wide range of confidence intervals and significant differences between studies visually confirm a high level of heterogeneity (I^2^ = 71.9%). Studies with wide intervals indicate uncertainty or small samples, while narrow intervals suggest more stable results. (**a**) Shows significant differences among highly relevant algorithms, with wide confidence intervals; (**b**) Shows less variability among algorithms with partial or low relevance.

For visual comparison (Figure 4), mean efficiency, standard deviation, and sample size were extracted for each algorithm across all contributing studies. Confidence intervals were calculated using aggregated estimates rather than single-study values, and no simulated data were used.

## 3. Results

### 3.1. Algorithm Performance

Across all studies, the weighted average accuracy of multimodal algorithms was 90.2% (±5.0%), indicating strong average performance but substantial inter-study variability (I^2^ = 71.9%). The variability (±5.0%) reflects standard deviation across studies rather than measurement precision. The highest-performing methods (>91%) included:

1. Neural Transformers (93.5%)—highest accuracy, but computationally demanding.
2. GANs (93.2%)—strong image generation, limited by stability.
3. Multimodal Fusion (92.3%), Dynamic Connectivity Models (92.0%), Supervised/Deep Learning, and Real-Time Feedback Systems (all ~91%).

Algorithms like Dynamic Connectivity Models (DCM) and Independent Component Analysis (ICA) consistently showed robust efficiency and are highly translatable (Table 1, Figure 2).

Reported accuracy values (e.g., 91–94%) reflect performance within the original study settings and were not benchmarked against intraoperative electrocorticography, direct cortical stimulation, or other gold-standard modalities. These values should therefore be interpreted as relative rather than confirmatory indicators of clinical accuracy [10,15,16,17,18,19,20]

Detailed analysis is available in the Supplementary Materials (Supplementary Results) (Table S2).

### 3.2. Trade-Off Between Efficiency and Processing Time

Efficiency gains often came at the cost of longer processing times (Figure 3). Processing times mentioned in the text represent average per-iteration latency values reported in the primary studies (expressed in milliseconds), whereas Figure 3 illustrates total algorithm runtime normalized to seconds for comparability across methods.

Bayesian Fusion, ICA, and CCA balanced accuracy and speed (processing ~100–125 ms per processing cycle), demonstrated the most favorable latency–accuracy balance among the evaluated methods, suggesting potential suitability for intraoperative adaptation pending further clinical validation [21].

DCM and Transformers offered very high accuracy but slower processing (140–180 ms per processing cycle), which may reduce feasibility in applications requiring real-time or iterative feedback rather than affecting data acquisition itself.

Although the acquisition of fMRI or EEG data occurs over minutes, these latency values refer to post-acquisition processing and are critical in iterative or closed-loop tasks where cumulative delays can impair time-sensitive decision-making.

GANs and Deep Learning showed promise but require computational optimization.

Recommendation: Prioritize Bayesian Fusion and ICA-based pipelines for real-time neurosurgical integration [22].

Detailed analysis is available in the Supplementary Materials (Supplementary Results) (Table S3).

### 3.3. Heterogeneity and Subgroup Analysis

Significant heterogeneity was observed (I^2^ = 71.9%).

Algorithms were stratified into relevance categories (highly vs. partially relevant) based on predefined clinical translation criteria, including: (1) validation in presurgical or intraoperative settings, (2) feasibility for real-time or near-real-time processing, (3) integration with stereotactic planning or navigation tools, (4) use in ≥3 independent clinical cohorts, and (5) demonstrated applicability for DBS targeting, functional mapping, or tumor margin assessment. Algorithms lacking clinical deployment, restricted to experimental datasets, or unsuitable for surgical workflows were classified as partially or low relevance. These criteria were directly applied in Table 2 when assigning algorithms to ‘Highly Relevant’ or ‘Partially/Low Relevant’ categories.

Highly relevant algorithms: I^2^ = 79.6% (greater variability across implementations). This high heterogeneity reflects methodological diversity among advanced algorithmic implementations rather than inconsistency in outcome direction. Subgroup analysis was therefore interpreted descriptively to identify general trends across algorithm families.

Partially relevant algorithms: I^2^ = 51.0% (more methodological consistency, but less clinical impact).

As a full meta-analysis was not feasible, results were synthesized qualitatively (Table 2, Figure 4). Given the high heterogeneity (I^2^ = 71.9%), a structured narrative synthesis was applied in place of quantitative pooling. Subgroup analysis was exploratory and aimed at identifying variability patterns rather than reducing heterogeneity below inferential thresholds.

Studies were compared within subgroups based on algorithm family and clinical application, and performance metrics were summarized using weighted means and value ranges rather than pooled effect sizes. Interpretation was informed by sample size and QUADAS-2 ratings, and no significance-based vote-counting was used. This approach allowed clinically meaningful comparisons without compromising methodological transparency. Interpretation of subgroup results reflects indicative trends rather than pooled statistical certainty.

To further explore whether performance differed between highly and partially relevant algorithms, group-level comparisons were conducted using ANOVA, *t*-tests, Mann–Whitney U, and Kruskal–Wallis tests on aggregated performance metrics. Exact *p*-values were reported to ensure transparency (ANOVA: *p* = 0.18; Mann–Whitney U: *p* = 0.27; Kruskal–Wallis: *p* = 0.34; *t*-test: *p* = 0.12). Corresponding 95% confidence intervals for mean differences are provided in the Supplementary Materials. These exploratory analyses revealed no statistically significant differences between relevance groups, indicating that performance variability primarily reflects methodological rather than outcome-related heterogeneity [23,24,25,26].

### 3.4. Risk of Bias

Bias assessment (QUADAS-2) showed:

Low risk studies (n = 12): highest average performance (~90%).

Moderate risk (n = 7): reduced accuracy (~85%).

High risk (n = 4): lowest performance (~77%) [24,25].

Although correlations between risk domains and performance were weak (all *p* > 0.05), a trend toward lower efficiency with higher bias was evident.

Detailed information is available in the Supplementary Materials (Supplementary Results) (Figures S1–S4; Text S1).

### 3.5. Statistical Comparisons

To explore whether efficiency differed between relevance subgroups, we conducted descriptive and inferential comparisons using aggregated performance metrics (ANOVA, *t*-test, Mann–Whitney U, and Kruskal–Wallis), as detailed in the Supplementary Materials. None of the tests showed statistically significant differences between highly relevant and partially relevant algorithms (all *p* > 0.05). This indicates that the small observed variability reflects methodological heterogeneity across studies rather than true differences in algorithmic efficacy.

Detailed information is available in the Supplementary Materials (Supplementary Results) (Figure S5; Text S2).

### 3.6. Publication Bias

Publication bias was assessed using both visual and statistical approaches to ensure the robustness of the findings. Funnel plots were first inspected to detect potential asymmetry, followed by Egger’s regression test to quantify small-study effects.

Egger’s test (*p* = 0.76) and funnel plots indicated no significant publication bias. Trim-and-fill analysis confirmed robustness of pooled results.

Detailed information is available in the Supplementary Materials (Supplementary Results) (Figures S6 and S7; Text S3).

### 3.7. Key Determinants of Algorithm Performance

Computational complexity: DCMs and Transformers achieve the highest accuracy but require large-scale GPU/TPU resources.

Artifact removal: Advanced ICA and CCA methods improve EEG-fMRI integration, while simpler filters yield weaker results.

Clinical validation: Only a minority of studies included >50 patients. Most remain experimental, underscoring the need for multicenter trials.

Summary: While high accuracy is achievable (90–94%), real-time clinical translation depends on optimization of computational load, artifact handling, and validation on large patient datasets.

Detailed information is available in the Supplementary Materials (Supplementary Results) (Tables S4–S7; Text S4).

## 4. Discussion

This systematic review critically examined computational approaches for integrating fMRI and EEG in stereotactic neurosurgery. While many algorithms demonstrate high performance in controlled or experimental settings, evidence for clinical readiness remains limited. Major barriers include methodological heterogeneity, reliance on small or simulated datasets, and inconsistent reporting of preprocessing and validation steps [20]. These issues reduce reproducibility and may inflate reported accuracy.

### 4.1. Algorithm-Specific Advantages, Limitations, and Technical Features of Multimodal Data Integration Algorithms

A detailed analysis of the algorithms reviewed reveals distinct advantages, disadvantages, and technical considerations that determine their clinical translatability.

#### 4.1.1. Dynamic Connectivity Models (DCM) and Bayesian Fusion

These model-based approaches provide high interpretability and strong accuracy (≈92–94%), enabling detailed mapping of directed connectivity. Their advantages lie in robustness to missing data and probabilistic inference. However, their disadvantages include high computational cost, parameter complexity, and relatively slow runtime (≈140–180 ms per iteration), limiting real-time intraoperative application.

These methods rely on generative models that estimate latent neural states and their coupling to fMRI signals. Bayesian approaches explicitly model uncertainty and prior distributions, while DCM estimates effective connectivity by fitting a biophysical model to multimodal time series. Integration is achieved through hierarchical inference, allowing simultaneous evaluation of neural dynamics and hemodynamic responses [21].

#### 4.1.2. Independent Component Analysis (ICA) and Canonical Correlation Analysis (CCA)

Data-driven decomposition methods achieve robust performance (≈91–92%) with relatively low latency (≈100–125 ms). They are advantageous for noise separation and rapid computation, making them practical for intraoperative workflows. Their main disadvantages are sensitivity to artifacts and limited ability to capture nonlinear interactions, which can reduce generalizability.

These decomposition methods operate on feature-level fusion. ICA separates statistically independent sources across modalities, while CCA maximizes correlations between EEG and fMRI features [22].

#### 4.1.3. Joint ICA (jICA) and Multimodal Fusion

These hybrid methods provide comprehensive brain mapping across modalities, with strong integrative power (≈92–93%). Their advantages are broad applicability and stable accuracy across different datasets. However, they require complex calibration and preprocessing, and they carry higher computational demands compared to ICA/CCA alone.

jICA extends ICA to jointly estimate components across datasets. Such approaches are efficient and provide interpretable spatial–temporal patterns, though they assume linear mixing models and may be less suited for nonlinear dynamics.

These frameworks combine feature spaces or latent representations using factorization, regression, or weighted averaging strategies. By integrating complementary information across modalities, they enhance spatial resolution from fMRI and temporal resolution from EEG. However, their accuracy depends heavily on preprocessing pipelines and normalization procedures [23].

#### 4.1.4. Neural Transformer Models and Deep Learning Architectures (e.g., GANs, CNNs)

These AI-driven techniques achieve high reported accuracy (≈93–94%) in retrospective or simulated datasets. However, these figures have not been benchmarked against intraoperative electrocorticography or other clinical gold standards, limiting their proven applicability in real-world surgical settings. Their advantages are scalability and the ability to discover hidden patterns beyond traditional statistical models. Disadvantages include dependence on large datasets, computational intensity, training instability (especially for GANs), and limited interpretability. Current feasibility in intraoperative use is therefore constrained.

Neural networks achieve data-level fusion by jointly learning representations from EEG and fMRI inputs. CNNs extract spatial–temporal features, Transformers capture long-range dependencies with attention mechanisms, and GANs generate synthetic multimodal data to improve robustness. Efficiency, defined here as inference latency per sample (≈140–180 ms for Transformers) and computational resource requirements, is considered separately from predictive accuracy, as high accuracy does not necessarily imply real-time intraoperative feasibility. These approaches are powerful but require large training datasets, high-performance computing, and careful tuning to avoid overfitting. Future studies should validate these models against clinical gold standards to establish their translational potential and refine real-time intraoperative pipelines [24].

#### 4.1.5. Real-Time Feedback Systems and Online Machine Learning

These lightweight, adaptive models provide immediate responses (≈100–125 ms), aligning well with intraoperative monitoring and neurofeedback. Advantages include high adaptability and integration potential with surgical navigation. However, disadvantages are somewhat lower peak accuracy compared to deep learning, and vulnerability to noise during live acquisition.

These systems emphasize adaptive, closed-loop integration. EEG features are aligned in real time with hemodynamic changes from fMRI, and machine learning models update iteratively during acquisition. This enables near-immediate feedback but often at the cost of reduced complexity compared to deep learning models [25].

#### 4.1.6. Graph-Theoretical and Temporal Analysis Models

These methods characterize large-scale brain networks and temporal dynamics. Their advantages lie in interpretability and the ability to highlight system-level interactions. However, they are computationally less efficient, less precise for localizing functional areas, and thus less suited for direct neurosurgical decision-making.

These techniques model EEG–fMRI integration at the network level. Graph methods identify nodes and edges across modalities, while temporal models evaluate dynamic connectivity over time. Their strength lies in characterizing large-scale network interactions, though they are computationally intensive and less precise for localized brain mapping.

Overall, accuracy–latency trade-offs remain central: algorithms with the highest accuracy (Transformers, GANs, DCM) require further optimization for real-time use, while ICA/CCA and real-time feedback systems represent the most feasible options for intraoperative application [26].

### 4.2. Clinical Implications

Algorithms balancing accuracy and processing speed (e.g., Bayesian Fusion, ICA pipelines) appear most translatable to intraoperative workflows. However, their applicability remains provisional and contingent on validation in prospective clinical studies, despite favorable computational performance. If validated, multimodal integration could enhance mapping of functionally critical cortical regions (cortical regions responsible for essential neurological functions), refine DBS targeting, and reduce invasive electrode use [22]. However, clinical translation requires validated real-time pipelines, integration into navigation systems, and demonstration of improved surgical outcomes in prospective trials [27].

### 4.3. Stereotactic Radiosurgery (SRS)

Although most current applications of EEG–fMRI integration focus on surgical planning and intraoperative guidance, its potential role in stereotactic radiosurgery (SRS) is increasingly discussed in the context of preserving functional networks near treatment targets.

Although fMRI–EEG integration is not standard in SRS planning, these techniques may help protect functional tissue near radiosurgical targets. Clinical application will require proof of spatial accuracy at radiotherapy planning resolution and reproducibility across platforms [1,2,3].

### 4.4. Recommendations and Future Directions

**Standardization:** Unified preprocessing pipelines and reporting checklists are essential to reduce heterogeneity.

**Validation:** Large multicenter trials with predefined endpoints (safety, decision-making, functional outcomes) are required.

**Optimization:** Lightweight, explainable models and GPU/TPU acceleration will improve real-time feasibility.

**Transparency:** Open sharing of datasets and code will support reproducibility and independent verification.

**Personalization:** Adaptive algorithms trained on heterogeneous data may enable patient-specific brain mapping.

### 4.5. Clinical and Infrastructural Challenges

From a practical neurosurgical perspective, the major limitation is not the algorithmic efficacy itself, but rather the accessibility and cost of advanced neuroimaging infrastructure.

Functional MRI remains an expensive, high-maintenance modality that is not routinely available in many neurosurgical or community hospital settings.

Consequently, the clinical translation of multimodal EEG–fMRI frameworks depends heavily on institutional resources, technical expertise, and infrastructure support.

Future development should therefore focus on scalable and cost-effective approaches, including portable or simplified functional imaging platforms, to make algorithm-based brain mapping feasible beyond highly specialized centers.

Emerging hybrid modalities, such as low-field portable MRI, wearable EEG–fNIRS systems, and cloud-based computational pipelines, represent promising directions toward making functional neuroimaging more practical, affordable, and accessible in daily neurosurgical workflow [23,24,25].

### 4.6. Limitations

This review included only peer-reviewed, indexed studies, excluding preprints. Heterogeneity (I^2^ = 71.9%) limited quantitative synthesis, and most algorithms remain insufficiently validated in clinical settings. Notably, reported high accuracies for algorithms such as Neural Transformers, GANs, and DCM are based on retrospective or simulated datasets and have not been benchmarked against intraoperative gold standards, such as electrocorticography (ECoG), limiting their proven applicability in real-world surgical contexts.

Additionally, efficiency metrics—defined here as inference latency per sample and computational resource requirements—are reported separately from predictive accuracy, and high reported accuracy does not necessarily imply real-time intraoperative feasibility.

Findings should therefore be interpreted cautiously until larger-scale, prospective clinical validation studies are available.

### 4.7. Overall Perspective

Despite current limitations, multimodal AI-driven approaches show transformative potential for neurosurgery. Highly relevant algorithms such as DCM, CCA, and Multimodal Fusion provide strong accuracy but require further optimization for real-time use [16,18]. By contrast, Bayesian Fusion and ICA offer a practical balance of performance and feasibility [21]. Future progress will depend on interdisciplinary collaboration to refine models, expand clinical validation, and establish robust real-time applications for stereotactic neurosurgery. Unlike previous reviews that mainly summarized technical progress, this study provides a cross-comparative framework linking computational performance with real clinical utility, thus bridging the gap between algorithmic development and neurosurgical practice.

## 5. Conclusions

This systematic review highlights the promise of EEG–fMRI integration for stereotactic neurosurgery, while underscoring the current gap between experimental performance and clinical readiness.

Bayesian Fusion and ICA-based pipelines emerge as the most translatable approaches for real-time use, offering a practical balance of accuracy and computational speed [19,21]. By contrast, Neural Transformers and DCM achieve higher accuracy but remain limited by computational complexity and lack of clinical validation [16,18].

Key challenges include: inconsistent preprocessing pipelines and artifact removal, high computational demands, and limited validation in large, prospective patient cohorts [20,23].

Future progress requires standardized methodologies, multicenter clinical trials, and computational optimization through GPU/TPU acceleration. Interdisciplinary collaboration between clinicians, engineers, and data scientists will be essential to translate these algorithms into routine surgical practice [24,25,26,27].

Ultimately, multimodal AI-driven models hold the potential to enhance neurosurgical precision, reduce risks, and enable patient-specific interventions. Addressing current limitations will determine whether these promising technologies can transition from the laboratory into widespread clinical application.

## Figures and Tables

**Table 1:** Qualitative synthesis of algorithms *.

Algorithm Type	Specific Application	Challenges	Strengths	Weaknesses	Success Factors/Accuracy	Efficiency/Latency
Neural Transformer Models	Presurgical network prediction and fusion of EEG-fMRI during targeting	High computational load and large-scale multimodal data requirements	High precision and scalability	Limited interpretability and dependency on pretrained architectures	Simultaneous EEG–fMRI datasets (*n* ≥ 30); accuracy based on retrospective/simulated data, not benchmarked against intraoperative gold standards (e.g., ECoG)	≈140–180 ms per sample; high GPU/TPU usage
Dynamic Connectivity Models (DCM)	Mapping dynamic brain networks	Complex model specification and parameter tuning	Detailed network mapping	Reduced robustness in noisy or incomplete datasets	Tuned Bayesian/DCM model parameters; accuracy from controlled studies, not clinical gold standards	≈140–180 ms per iteration; high computational cost
Independent Component Analysis (ICA)	Noise reduction, component separation	Requires extensive preprocessing and artifact correction	Effective noise separation	Residual noise can impair source separation and accuracy	Artifact-filtered EEG inputs; accuracy from experimental datasets	≈100–125 ms per sample; low–moderate computational demand
Canonical Correlation Analysis (CCA)	Correlation of EEG and fMRI signals	Limited scalability for large multimodal datasets	Enhanced signal correlation	Inability to fully capture nonlinear brain signal relationships	Multimodal signal modeling frameworks; accuracy not benchmarked intraoperatively	≈100–125 ms per sample; low–moderate computational demand
Real-time Machine Learning	Adaptive learning for brain state prediction	High training data demands and parameter optimization	Fast adaptability	Performance degradation when datasets are small or imbalanced	Real-time GPU/TPU-based processing; accuracy experimental only	≈100–125 ms per sample; low-latency pipelines
Joint ICA (jICA)	Enhanced source localization	Challenging calibration across subjects and modalities	Improved source localization	Sensitive to misalignment and preprocessing variability	Validated ICA/jICA calibration protocols; accuracy from retrospective datasets	≈120–150 ms per sample; moderate computational demand
AI Integration Models	Integrated AI for complex data	Complex data fusion and multi-layer optimization	Complex data handling	Low model interpretability and risk of overfitting	Transparent parameter-based inference	≈140–180 ms per iteration; high computational cost
Multimodal Fusion	Fusion of EEG, fMRI, and DTI	Integration of heterogeneous signals across acquisition platforms	Comprehensive brain mapping	High computational and memory demands	Combined EEG–fMRI–DTI workflows; accuracy not clinically benchmarked	≈130–160 ms per sample; high memory requirements
Bayesian Models	Probabilistic brain activity mapping	Intensive calibration and convergence management	Robust probabilistic modeling	Lower transparency in posterior probability interpretation	Bayesian posterior verification; accuracy from controlled datasets	≈120–160 ms per sample; moderate computational load
Generative Adversarial Networks (GANs)	High-resolution brain image generation	Computational burden and tuning instability	Realistic brain representations	Mode collapse and inconsistent convergence	High-fidelity neural data generators (GAN-based); accuracy from simulations	≈150–200 ms per sample; high GPU usage
Temporal Analysis Models	Detection of brain state dynamics	Balancing time resolution and data volume	Capturing dynamic changes	Lower spatial accuracy in rapidly changing states	Online temporal resolution adjustment; accuracy not clinically validated	≈120–150 ms per sample; moderate computational demand
Hybrid Statistical Models	Blending statistical approaches	Integration of statistical and machine learning pipelines	Flexible integration	Vulnerable to overfitting when datasets are small	Hybrid statistical–ML frameworks; accuracy not benchmarked intraoperatively	≈130–180 ms per sample; high GPU/TPU usage
Advanced ICA	Advanced noise filtering	Requires high-fidelity denoising and structured preprocessing	Noise resilience	Susceptibility to latent artifacts and component misclassification	Noise-filtered ICA pipelines; accuracy from controlled datasets	≈120–160 ms per sample; moderate computational load
Supervised Learning Models	Accurate classification of brain states	Extensive labeled data requirements	High accuracy	Reduced generalizability outside training cohorts	Large annotated clinical datasets; accuracy not benchmarked intraoperatively	≈130–180 ms per sample; high GPU/TPU usage
Signal Decoding Models	Decoding neural signals	Managing interference and signal separation	Detailed signal decoding	Lower precision in noisy neural environments	Optimized EEG-fMRI preprocessing; accuracy from simulated/retrospective datasets	≈100–130 ms per sample; real-time pipelines
Dynamic Network Models	Modeling dynamic network activity	Complexity in modeling evolving neural connections	Capturing network dynamics	Reduced clarity in clinical interpretation	Parallelizable network models	≈140–180 ms per iteration; high computational cost
Real-time Feedback Systems	Real-time surgical feedback	Dependence on synchronized hardware and software platforms	Immediate clinical feedback	Limited adaptability to intraoperative signal variability	Intraoperative feedback compatibility	≈100–125 ms per sample; low-latency pipelines
Neurofeedback Algorithms	Optimizing neurofeedback	Calibration of personalized input-output loops	Enhanced learning efficiency	Variability in clinical responsiveness and outcome tracking	Closed-loop clinical neurofeedback; accuracy from simulated/retrospective datasets	≈100–130 ms per sample; real-time pipelines
Deep Learning Models	Complex pattern recognition	High GPU/TPU resource requirements	Advanced pattern detection	Black-box decision processes and limited transparency	Optimized inference pipelines; accuracy not benchmarked intraoperatively	≈130–180 ms per sample; high GPU/TPU usage
Dynamic Causal Modeling	Functional connectivity modeling	Intensive parameter estimation and customization	Detailed connectivity modeling	Difficulty adapting to diverse clinical datasets	Adaptive connectivity optimization	≈140–180 ms per iteration; high computational cost
Graph Theory Models	Analyzing brain networks	Complex computation and network reconstruction	Complex network analysis	Limited validation for surgical workflows	Clinically benchmarked graph analytics	≈130–160 ms per sample; high computational demand
Bayesian Fusion	Probabilistic data integration	High model variance and training time	Adaptive data modeling	Variable accuracy across heterogeneous cohorts	Cross-cohort reproducibility	≈120–160 ms per sample; moderate computational load
CCA + Time-Frequency Analysis	Temporal and frequency domain analysis	Managing multimodal and multiscale integration	Multidimensional analysis	Trade-offs in resolution and analysis depth	Time–frequency–connectivity fusion; accuracy based on retrospective/simulated data, not benchmarked against intraoperative gold standards (e.g., ECoG)	≈100–125 ms per sample; moderate computational demand

* Reported accuracy and success factors are based on retrospective or simulated datasets. They have not been validated against intraoperative gold standards such as electrocorticography (ECoG). Efficiency metrics indicate inference latency per sample and computational resource requirements, and are reported separately from predictive accuracy.

**Table 2:** Relevance of algorithms for stereotactic neurosurgery *.

Algorithm	Relevance Level	Clinical Application	Limitations
Dynamic Connectivity Models (DCM)	Highly Relevant	Real-time brain network mapping	Complex modeling
Canonical Correlation Analysis (CCA)	Highly Relevant	Localization of functional brain regions	Linear relationship limitation
Independent Component Analysis (ICA)	Highly Relevant	Noise separation and signal enhancement	Noise sensitivity
Joint ICA (jICA)	Highly Relevant	Source localization	Complex calibration
Multimodal Fusion	Highly Relevant	Comprehensive brain mapping	High processing demands
Bayesian Models	Highly Relevant	Probabilistic brain activity mapping	Model interpretability
Neural Transformer Models	Highly Relevant	Advanced data processing	High computational load
Real-time Feedback Systems	Highly Relevant	Real-time surgical feedback	Integration with clinical tools
Generative Adversarial Networks (GANs)	Highly Relevant	High-resolution brain data generation	Training instability
Deep Learning Models	Partially Relevant	Pattern recognition in brain signals	Data-intensive training
Hybrid Statistical Models	Partially Relevant	Combining statistical and ML methods	Limited clinical validation
Dynamic Network Models	Partially Relevant	Dynamic brain network analysis	Processing complexity
Signal Decoding Models	Low Relevance	Neural signal decoding	Limited surgical application
Temporal Analysis Models	Low Relevance	Temporal pattern analysis	Low precision for localization
Graph Theory Models	Low Relevance	Global brain network analysis	Not suited for surgery
Neurofeedback Algorithms	Low Relevance	Therapeutic applications, not surgical	Limited to therapy, not surgery

* Relevance levels in the table reflect predefined clinical translation criteria (validation in ≥ 3 cohorts, surgical feasibility, and real-time applicability).

## Data Availability

No new data were created or analyzed in this study.

## Supplementary Materials

The following supporting information can be downloaded at: https://www.mdpi.com/article/10.3390/brainsci15111188/s1, Table S1: Summary of EEG–fMRI integration studies analyzed in this review; Table S2: Detailed Analysis of Algorithms With 95% CI; Table S3: Algorithm analysis with additional metrics; Text S1: Risk of bias and its impact on algorithm performance; Figure S1: Rows represent individual studies; Figure S2: Correlation of risk domain with algorithm performance; Figure S3: Horizontal bar chart; Figure S4: Scatter plot with a regression line that visualizes the connection between the risk of bias and the efficiency of the algorithms; Text S2: Metaregression and statistical comparisons; Figure S5: Left graph—Violin Plot (Mann-Whitney U Test); Text S3: Consideration of publication bias and risk of bias; Figure S6: Funnel Plot—assessment of bias in the publication of studies; Figure S7: Results of Duval and Tweedie Trim-and-Fill Analysis; Text S4: Recommendations for optimization of algorithms for integration of FMRI and EEG in stereotactic neurosurgery; Table S4: Highly relevant algorithms-challenges and recommendations for optimization; Table S5: Partially relevant algorithms; Table S6: Low relevance algorithms; Table S7: A detailed implementation plan for algorithm optimization in clinical practice.

## Abbreviations

The following abbreviations are used in this manuscript:

AI	Artificial Intelligence
ANOVA	Analysis of Variance
CCA	Canonical Correlation Analysis
CI	Confidence Interval
DBS	Deep Brain Stimulation
DCM	Dynamic Causal/Connectivity Modeling
DTI	Diffusion Tensor Imaging
EEG	Electroencephalography
fMRI	Functional Magnetic Resonance Imaging
GANs	Generative Adversarial Networks
GPU	Graphics Processing Unit
ICA	Independent Component Analysis
jICA	Joint Independent Component Analysis
ML	Machine Learning
PET	Positron Emission Tomography
PRISMA	Preferred Reporting Items for Systematic Reviews and Meta-Analyses
QUADAS-2	Quality Assessment of Diagnostic Accuracy Studies 2
SRS	Stereotactic Radiosurgery
TPU	Tensor Processing Unit
