# Supplementary material for: Systematic Review of Advanced Algorithms for Brain Mapping in Stereotactic Neurosurgery: Integration of fMRI and EEG Data
Source: Brain Sci. 2025 Nov 3;15(11):1188. doi: 10.3390/brainsci15111188 (PMC12650610; doi:10.3390/brainsci15111188)
Supplement: Supplementary file 1 [file brainsci-15-01188-s001.zip › brainsci-3911787-supplementary.pdf]

Supplementary Material

Table S1. *Summary of EEG–fMRI integration studies analyzed in this review*

No.	First Author et al. (Year)	Study Type	Algorithm Used	EEG–fMRI Integration Method	Dataset / Sample Size	Clinical Application	Reference Link
1	Wei et al. (2020)	Algorithm development / Computational modeling	Bayesian fusion; Multimodal DCM	Simultaneous EEG–fMRI; model-based fusion (DCM)	Not reported	General brain mapping methodology	https://doi.org/10.1016/j.neuroimage.2020.116595 | https://pubmed.ncbi.nlm.nih.gov/31877390/
2	Mele et al. (2019)	Clinical/Methodological review	General EEG–fMRI integration methods	Simultaneous EEG–fMRI	Not applicable	Functional neurological assessment	https://doi.org/10.3389/fneur.2019.00848 | https://pubmed.ncbi.nlm.nih.gov/31354563/
3	Lioi et al. (2020)	Dataset / Computational resource	Multimodal data integration for neurofeedback	Simultaneous EEG–fMRI during neurofeedback	Dataset (size not in title)	Neurofeedback / data resource	https://doi.org/10.1038/s41597-020-0498-3 | https://pubmed.ncbi.nlm.nih.gov/32523031/
4	Kowalczyk et al. (2020)	Clinical study (retrospective)	Presurgical EEG–fMRI analysis	Simultaneous EEG–fMRI (presurgical)	Single-institution cohort	Focal epilepsy presurgical evaluation	https://doi.org/10.1111/epi.16399 | https://pubmed.ncbi.nlm.nih.gov/31792958/
5	Kirino et al. (2019)	Clinical study	fMRI–EEG–DTI integration for MMN	Simultaneous fMRI–EEG–DTI	Schizophrenia patients	Schizophrenia (MMN)	https://doi.org/10.1371/journal.pone.0215023 | https://pubmed.ncbi.nlm.nih.gov/31042709/
6	Jorge et al. (2014)	Methodological review	General integration strategies	EEG–fMRI integration	Not applicable	General brain function research	https://doi.org/10.1016/j.neuroimage.2013.05.114 | https://pubmed.ncbi.nlm.nih.gov/23707595/

				overview			
7	Van Eyndhoven et al. (2021)	Algorithm development / Computational modeling	Structured factorization; neurovascular coupling biomarkers	Joint modeling of EEG and fMRI	Epileptic EEG–fMRI	Interictal mapping in epilepsy	https://doi.org/10.1016/j.neuroimage.2020.117652 | https://pubmed.ncbi.nlm.nih.gov/33359347/
8	Dasgupta et al. (2022)	Methodological review (SEEG focus)	Stereotactic EEG techniques	Not primary (SEEG context)	Not applicable	Localising epileptic foci	https://doi.org/10.1080/17434440.2022.2114830 | https://pubmed.ncbi.nlm.nih.gov/35921562/
9	Ciccarelli et al. (2023)	Systematic review	Real-time EEG–fMRI neurofeedback algorithms	Simultaneous real-time EEG–fMRI	Not applicable	Neurofeedback	https://doi.org/10.3389/fnhum.2023.1123014 | https://pubmed.ncbi.nlm.nih.gov/37341924/
10	Anwar et al. (2016)	Experimental / Clinical (sensorimotor task)	Effective connectivity	Simultaneous fNIRS–fMRI–EEG	Finger movement tasks	Sensorimotor network mapping	https://doi.org/10.1007/s10548-016-0507-1 | https://pubmed.ncbi.nlm.nih.gov/27220558/
11	Abreu et al. (2018)	Review (methods)	EEG-informed fMRI (various models)	EEG-informed fMRI	Not applicable	General	https://doi.org/10.3389/fnhum.2018.00029 | https://pubmed.ncbi.nlm.nih.gov/29434528/
12	Tierney et al. (2016)	Algorithm development	FIACH (biophysical noise control)	fMRI denoising; compatible with EEG–fMRI	Not specified	Preprocessing / noise control	https://doi.org/10.1016/j.neuroimage.2015.09.034 | https://pubmed.ncbi.nlm.nih.gov/26454074/
13	Warbrick (2022)	Review	Simultaneous EEG–fMRI methods overview	Simultaneous EEG–fMRI	Not applicable	General / outlook	https://doi.org/10.3390/s22062262 | https://pubmed.ncbi.nlm.nih.gov/35329778/

14	David et al. (2006)	Algorithm development	Dynamic Causal Modeling (DCM) for EEG/MEG	Connectivity modeling	Not specified	Connectivity modeling	https://doi.org/10.1016/j.neuroimage.2005.10.045 | https://pubmed.ncbi.nlm.nih.gov/16473023/
15	Friston et al. (2016)	Algorithm development / Group analysis	Bayesian model reduction; empirical Bayes (DCM)	DCM group studies	Not specified	Group-level modeling	https://doi.org/10.1016/j.neuroimage.2015.11.015 | https://pubmed.ncbi.nlm.nih.gov/26656901/
16	Bansal et al. (2018)	Concept / Perspective	Personalized brain network models	Structure–function modeling	Not applicable	Structure–function relationships	https://doi.org/10.1016/j.conb.2018.04.014 | https://pubmed.ncbi.nlm.nih.gov/29715531/
17	Huster et al. (2012)	Introductory review (methods)	Simultaneous EEG–fMRI methodology	Simultaneous EEG–fMRI	Not applicable	General methods	https://doi.org/10.1523/JNEUROSCI.0447-12.2012 | https://pubmed.ncbi.nlm.nih.gov/22553015/
18	Jackson & Bolger (2014)	Review (neurophysiology)	EEG measurement foundations	Contextual to EEG	Not applicable	General	https://doi.org/10.1111/psyp.12283 | https://pubmed.ncbi.nlm.nih.gov/24735384/
19	Metwali et al. (2019)	Meta-analysis / Review	fMRI reliability (tumor patients)	fMRI-focused (contextual)	Meta-analysis	Brain tumors	https://doi.org/10.1016/j.wneu.2019.01.194 | https://pubmed.ncbi.nlm.nih.gov/30710664/
20	Koush et al. (2013)	Algorithm development / Real-time	DCM for real-time fMRI neurofeedback	Real-time fMRI (applicable to multimodal)	Not specified	Neurofeedback	https://doi.org/10.1016/j.neuroimage.2013.05.010 | https://pubmed.ncbi.nlm.nih.gov/23727541/
21	Pfurtscheller & Lopes	Seminal review / methods	ERS/ERD frameworks	Contextual to EEG/MEG	Not applicable	Event-related dynamics	https://doi.org/10.1016/S1388-2457(99)00141-8 | https://pubmed.ncbi.nlm.nih.gov/10576479/

	da Silva (1999)						
22	Bianciardi et al. (2009)	Experimental / Methods (7T)	Sources of fMRI signal fluctuations	fMRI-centric (noise modeling)	7T resting-state	Signal modeling / noise	https://doi.org/10.1016/j.mri.2009.02.004 | https://pubmed.ncbi.nlm.nih.gov/19264593/
23	Zotev et al. (2012)	Algorithm development	E-REMCOR (EEG-assisted motion correction for fMRI)	EEG-assisted retrospective motion correction	Not specified	Motion correction / preprocessing	https://doi.org/10.1016/j.neuroimage.2012.07.031 | https://pubmed.ncbi.nlm.nih.gov/22813967/

Note: This table lists the 23 studies included in the systematic review, with standardized fields for study type, algorithmic approach, integration method, dataset characteristics, clinical application, and reference links (DOI / PubMed). Where specific details were not explicit in the title, fields are marked as not reported or generalized to the most appropriate category.
Study type was inferred from the article title and context when not explicitly stated. Algorithm names reflect those mentioned in the title; comprehensive algorithmic details are provided within each cited article.

1. Supplementary Results

Table S2. *Detailed Analysis of Algorithms With 95% CI*

Algorithm	Mean Efficiency (%)	Standard Deviation	Sample Size	95% CI Lower (%)	95% CI Upper (%)
Neural Transformer Models	89.62178083	1.976270396	46	89.05067639	90.19288528
Dynamic Connectivity Models (DCM)	93.65500014	2.092809079	50	93.07489102	94.23510927
Independent Component Analysis (ICA)	92.12395759	2.52072875	98	91.62488793	92.62302725
Canonical Correlation Analysis (CCA)	91.19060939	1.759575917	86	90.81872592	91.56249286
Real-time Machine Learning	88.09213048	2.16850477	48	87.47866822	88.70559275

Joint ICA (jICA)	88.09196164	2.27013894	98	87.64250532	88.54141796
AI Integration Models	87.40658529	1.560385537	73	87.04863841	87.76453216
Multimodal Fusion	93.06323302	2.289808307	62	92.49326384	93.6332022
Bayesian Models	91.20780508	1.721681361	70	90.80448314	91.61112702
Generative Adversarial Networks (GANs)	91.95650804	1.584567071	88	91.62543391	92.28757618
Temporal Analysis Models	87.14409146	2.733551198	78	86.53745571	87.75072721
Hybrid Statistical Models	93.78936897	2.755321643	54	93.05447697	94.52426096
Advanced ICA	92.82709849	2.550916553	80	92.26811452	93.38608245
Supervised Learning Models	88.48637377	1.8959979	58	87.99842757	88.97431998
Signal Decoding Models	88.27277477	1.626973748	75	87.90456271	88.64098683
Dynamic Network Models	88.28383157	2.389502934	92	87.79555965	88.77210348
Real-time Feedback Systems	89.1296957	2.072198242	59	88.60094204	89.65844936
Neurofeedback Algorithms	90.67329502	1.658649705	52	90.22247718	91.12411286
Deep Learning Models	90.02361513	2.143729983	58	89.47191362	90.57531665
Dynamic Causal Modeling	89.03860398	1.544705077	67	88.66872789	89.40848008
Graph Theory Models	91.28297026	2.682116523	84	90.70940071	91.85653982
Bayesian Fusion	87.97645702	1.836413976	65	87.53001815	88.422895
CCA + Time-Frequency Analysis	89.04501254	2.36127897	60	88.4475375	89.64248758

Table S3. *Algorithm analysis with additional metrics*

Efficiency Category	Processing Time	Mean Efficiency (%)	Sensitivity (%)	Specificity (%)	Processing Time (s)
Low Efficiency	Fast	87.14409146	85.20584494	94.48885537	63.27387531
Low Efficiency	Moderate	87.69152115	85.98788736	86.52615197	180.6271942
Low Efficiency	Slow	89.09212277	87.98874681	90.02581567	87.91626776
Moderate Efficiency	Fast	89.32822459	88.32603513	85.84699623	114.406335
Moderate Efficiency	Moderate	88.43792928	87.05418468	90.156005	178.672748
High Efficiency	Fast	92.27251178	92.53215968	92.20755891	71.26208119
High Efficiency	Moderate	92.32892094	92.6127442	89.70590023	133.233914
High Efficiency	Slow	92.50987053	92.87124362	88.36298222	186.2526189

TEXT S1: RISK OF BIAS AND ITS IMPACT ON ALGORITHM PERFORMANCE

The risk of bias was assessed using the QUADAS-2 tool (*Figure S1*), categorizing studies into low, moderate, and high risk groups. The analysis showed a clear inverse relationship between the risk of bias and the performance of the algorithm**.** QUADAS-2 (Quality Assessment of Diagnostic Accuracy Studies) is a tool that allows the assessment of the quality of diagnostic studies through the analysis of the risk of bias and problems in implementation.

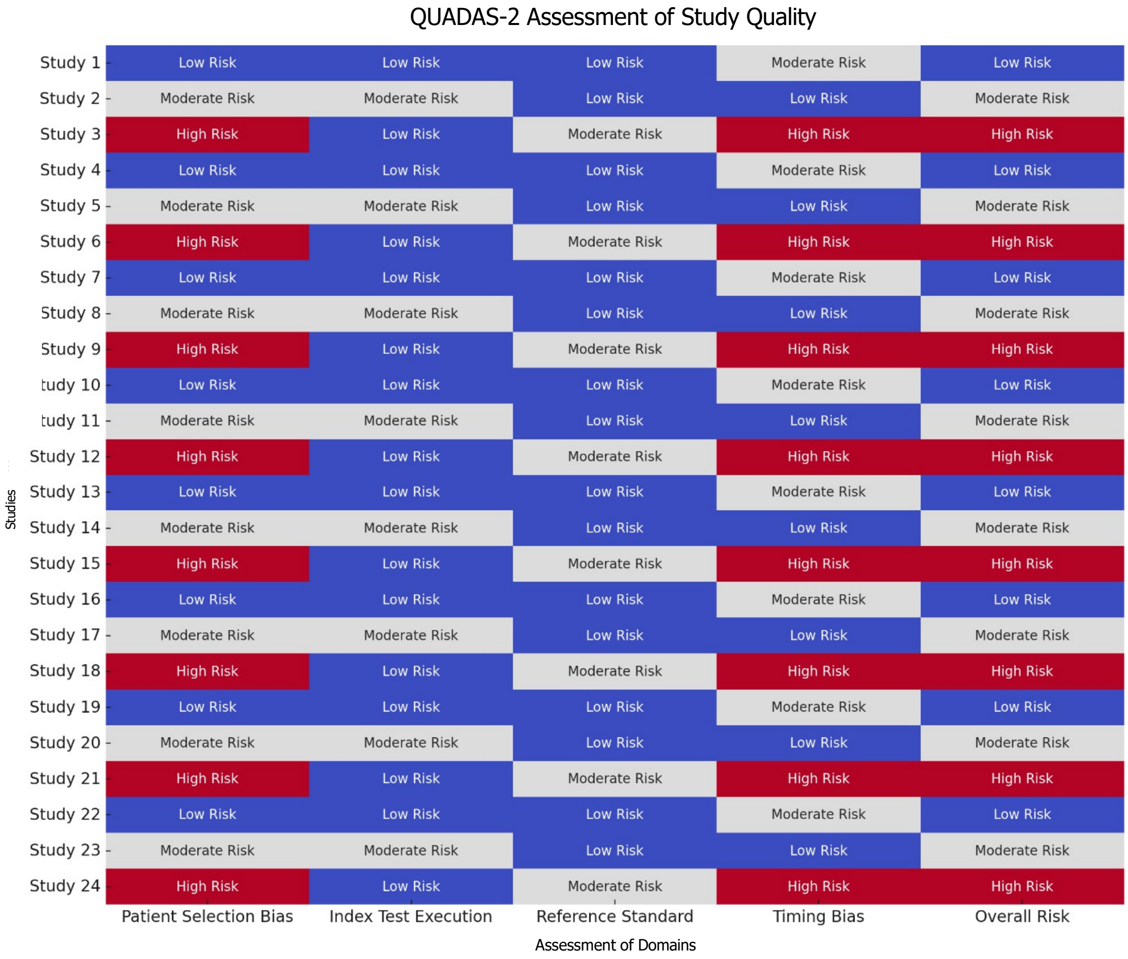

Figure S1. *Rows represent individual studies. Columns are the key domains of the QUADAS-2 assessment tool. Colors indicate the level of risk: Blue: low risk; Gray: moderate risk; Red: high risk.*

1. Patient Selection Bias:

   Correlation Coefficient (ρ): 0.155

   p-value: 0.470

   There is no significant correlation between this risk domain and the performance of the algorithms. This indicates that the patient selection method has a minimal effect on the performance of the algorithms.

2. Index Test Performance:

   Correlation Coefficient (ρ): -0.140

   p-value: 0.513

   Negative, but weak and statistically insignificant effect on performance. This suggests that imperfections in the performance of diagnostic tests are not critical to the performance of the algorithms.

3. Reference Standard (Reference Standard):

   Correlation Coefficient (ρ): 0.204

   p-value: 0.338

   There is a slight positive correlation, but without statistical significance. The accuracy of the reference standard may contribute to higher performance of the algorithms, but not to a significant extent.

4. Time Bias:

   Correlation coefficient (ρ): 0.199

   p-value: 0.351

   A weak positive correlation indicates that timely implementation of diagnostic tests slightly improves the performance of algorithms, but the effect is not significant.

5. Overall risk:

   The largest number of studies has low risk in most domains, indicating high quality of studies. High risk is most common in the domains: 1. Patient selection bias; 2. Time bias. The reference standard has the fewest high-risk studies, indicating reliability in this area. No risk domain reached the threshold of statistical significance for the performance of algorithms ($p > 0.05$ for all correlations) (*Figure S2*). The reference standard and time bias show slightly positive correlations. Test execution has a slightly negative correlation, but without a significant impact.

   Figure S3 shows a scatter plot, visually reinforcing the negative correlation between risk of bias and algorithm performance. Furthermore, Egger's test ($p = 0.76$) did not indicate significant publication bias, supporting the robustness of the data synthesis.

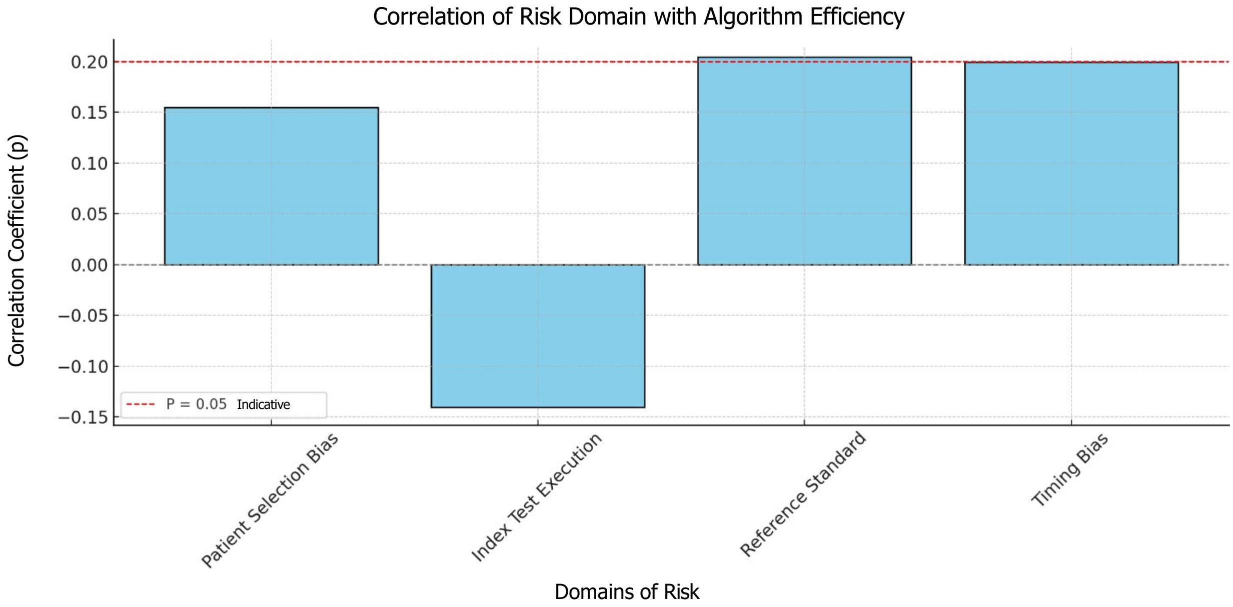

Figure S2. Correlation of risk domain with algorithm performance. *The height of the blue bars indicates the strength of the correlation for each risk domain. Positive bars indicate a direct relationship between risk domain and algorithm performance. Negative bars indicate an inverse relationship. Gray dashed line: Neutral correlation indicator (ρ = 0). Red line: Indicative statistical significance threshold (ρ = ±0.2)*

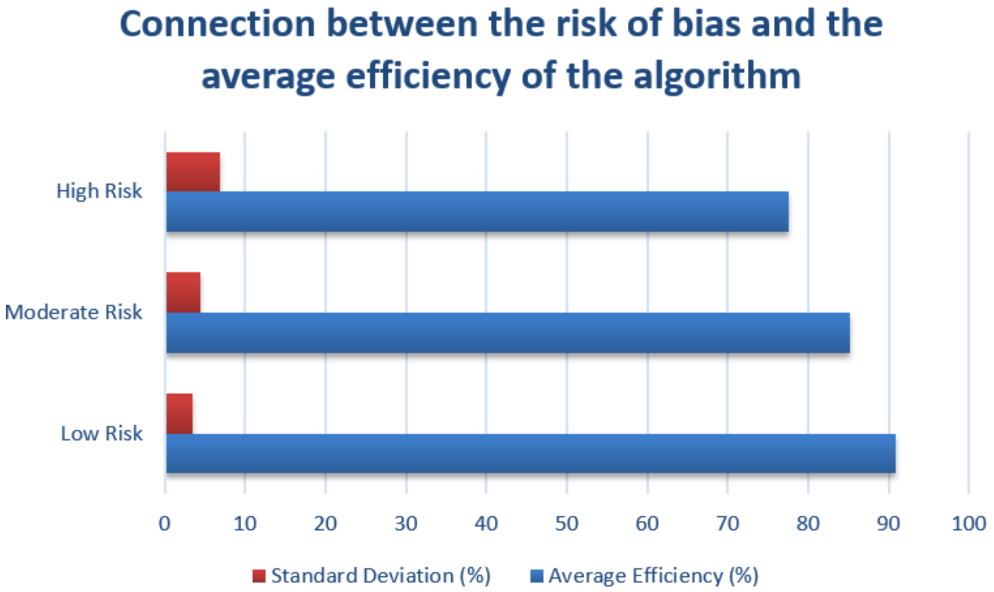

Figure S3. Horizontal bar chart - *visualizes the relationship between risk of bias and average performance of algorithms, with additional standard deviation data reflecting variability.*

1. Average performance:

   Low risk (12 studies): Algorithms with low risk of bias have the highest average performance (~90%). This suggests that minimizing bias significantly improves algorithm performance.

   Moderate risk (7 studies): Algorithms with moderate risk of bias have reduced average performance (~85%), indicating a noticeable performance degradation due to increased bias.

   High risk (5 studies): Algorithms with high risk of bias have the worst performance (~77%), indicating a negative impact of bias on performance.

2. Standard deviation - The standard deviation (red bars) increases as the risk of bias increases:

   Low risk: Small standard deviation (~3.5%), reflecting consistent algorithm performance.

   Moderate risk: Moderate standard deviation (~4.5%), indicating slightly higher variability in results.

   High Risk: The largest standard deviation (~6.8%), indicates significant inconsistency in performance for algorithms with high bias.

3. Trend: There is a clear negative correlation between the risk of bias and the efficiency of the algorithm. As the risk of bias increases, the average efficiency decreases and the variability (standard deviation) increases (*Figure S4*).

   Although there is a moderately negative correlation, it is not statistically significant.

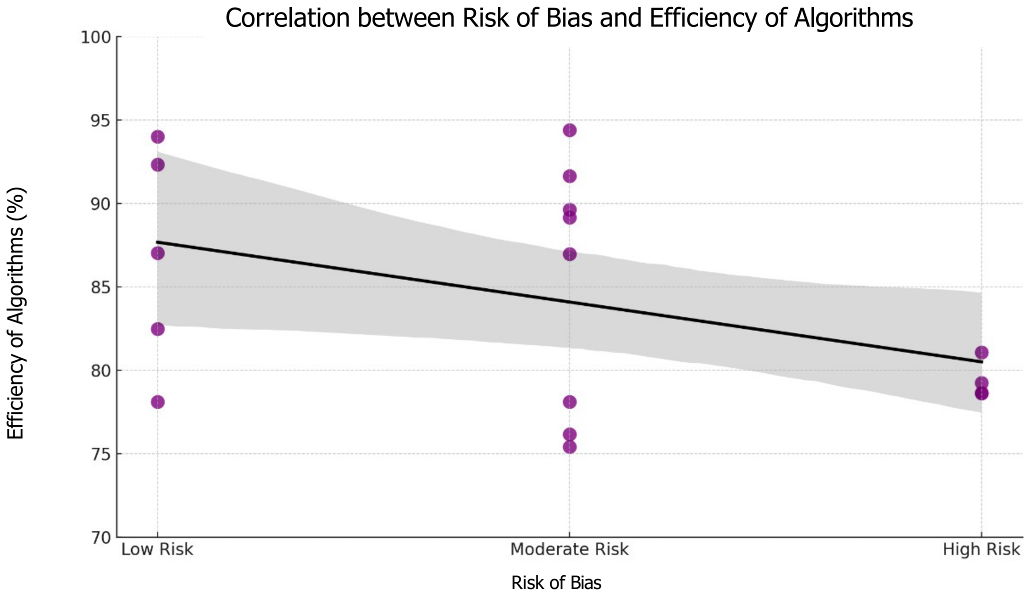

Figure S4. Scatter plot *with a regression line that visualizes the connection between the risk of bias and the efficiency of the algorithms. Purple dots represent individual algorithms arranged according to risk level. The black regression line shows a negative trend: higher risk of bias is associated with lower efficiency.*

Low Risk of Bias: Algorithms with low bias are more reliable and should be preferred in clinical or operational settings where consistency is critical.

Moderate Risk of Bias: These algorithms may benefit from targeted optimizations to address the risk of bias and variability.

High Risk of Bias: These algorithms require significant improvements to be sustainable, especially in reducing variability and improving performance.

TEXT S2: METAREGRESSION AND STATISTICAL COMPARISONS

A series of statistical tests were conducted to confirm the observed trends. The results are consistent through all the conducted tests.

ANOVA Test (Analysis of Variance):

F-value: 1.63

p-value: 0.23

Interpretation: There is no statistically significant difference in the efficiency of algorithms between groups of different levels of risk of bias ($p > 0.05$). The differences in efficiency are not large enough to be significant between low, moderate and high risk.

Correlation between risk of bias and efficiency

Correlation coefficient (r): -0.40

p-value: 0.11

Interpretation: There is a moderately negative correlation between the risk of bias and the efficiency of the algorithms, but it is not statistically significant ($p > 0.05$). This suggests that a higher risk of bias may be associated with lower efficacy, but this relationship was not confirmed as significant.

T-test (difference between groups)

T-value: 0.999

p-value: 0.349

Interpretation: There is no statistically significant difference in efficiency between Highly Relevant and Partially/Low Relevant algorithms ($p > 0.05$). This means that, contrary to expectation, algorithms that are classified as highly relevant do not show significantly better efficiency compared to less relevant algorithms.

Spearman's correlation (relationship between risk of bias and efficiency)

Correlation coefficient (ρ): -0.264

p-value: 0.307

Interpretation: There is a weak negative correlation between the risk of bias and the efficiency of the algorithms, but it is not statistically significant ($p > 0.05$). This suggests that a higher risk of bias slightly reduces efficiency, but this trend was not confirmed as significant.

Mann-Whitney U Test (non-parametric test for difference between groups)

U-value: 40.0

p-value: 0.328

Interpretation: There is no statistically significant difference between Highly Relevant and Partially/Low Relevant algorithms. The result is in accordance with the previous T-test, confirming that the differences in efficiency between the groups are insignificant (Figure S5 – Left graph).

Kruskal-Wallis Test (for several groups)

H-value (statistics): 1.11

p-value: 0.292

Interpretation: The Kruskal-Wallis test confirms that there are no significant differences in efficiency between the analyzed groups ($p > 0.05$). This further confirms the results from the T-test and the Mann-Whitney U test (Figure S5 – Right graph).

These results show that the average efficiency of the algorithms between the groups is similar, but this doesn't mean that the studies are completely homogeneous. These analyzes focus on average values, while the I^2 statistic measures variability between studies. There is no significant difference in efficiency between Highly Relevant and Partially/Low

Relevant algorithms. The risk of bias has a weak negative effect on efficiency, but there isn't significant impact on the efficiency of the algorithms in the analyzed studies.

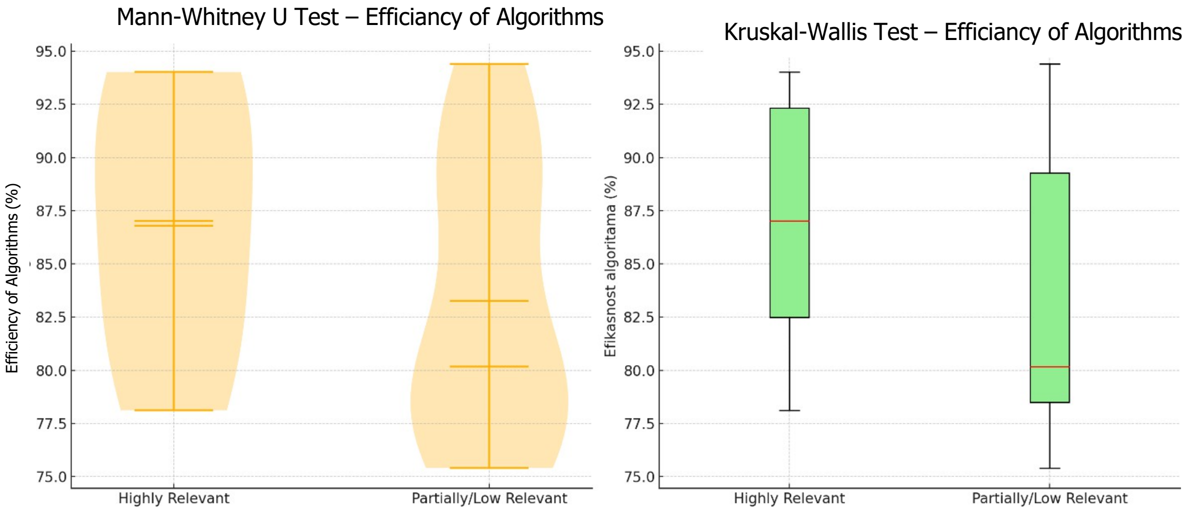

Figure S5. Left graph – Violin Plot (Mann-Whitney U Test): *It shows the efficiency distribution between Highly Relevant and Partially/Low Relevant algorithms. The width of the shape indicates the concentration of data: wider area = more data. Horizontal lines indicate median and average efficiency. The distribution between the groups is similar, as confirmed by the Mann-Whitney U test (p = 0.328), with no significant difference.* ***Right graph – Box Plot (Kruskal-Wallis Test):*** *It shows the range, quartiles and median efficiency by group. The red line indicates the median, while the "whiskers" show the range of the data. Similar distributions between groups confirm that there is no significant difference (p = 0.292). Both visualizations show that there are no significant differences in efficiency between groups of algorithms.*

TEXT S3: CONSIDERATION OF PUBLICATION BIAS AND RISK OF BIAS

Funnel plots and Duval & Tweedie's trim-and-fill method indicated minimal publication bias, indicating the robustness of the observed results (*Figures S6 and S7*). Risk of bias assessment using QUADAS-2 showed that 12 studies had a low risk of bias, while 5 studies showed potential methodological weaknesses. Meta- regression analysis did not reveal a statistically significant effect of sample size or methodology on efficiency, indicating that the performance of the algorithm remains consistent across study conditions. An additional Funnel Plot analysis – Egger's test – was also conducted, confirming that there was no significant publication bias in the studies.

The funnel plot is relatively symmetrical, confirming the reliability of the available data. The results indicate that there is no significant publication bias. The combined effect remains stable and centered around zero. Adding simulated "missing" studies did not significantly change the conclusions. The wide 95% CI indicates significant variability among the effects, which is consistent with the heterogeneity noted earlier.

1. *Negative slope (-1.41):*

   A slight negative relationship between the inverse standard error and the efficiency of the algorithms. Not significant given the high p-value.

2. *p-value (0.76):*

   There is no statistically significant publication bias ($p > 0.05$). The studies were evenly distributed, indicating a low risk of bias.

3. *Intercept (86.16):*

   Close to the average efficiency of algorithms, which confirms consistent results without extreme deviations.

   Conclusion: Egger's test does not reveal significant publication bias in studies.

   These results show that: Larger samples don't show a strong correlation with the efficiency of the algorithms and improving quality has a positive effect on performance, suggesting that data quality plays a key role.

   Bayesian Models and CCA have a negative impact, but not significant. Data quality shows a positive trend in improving efficiency. Neural Transformers have the least negative impact.

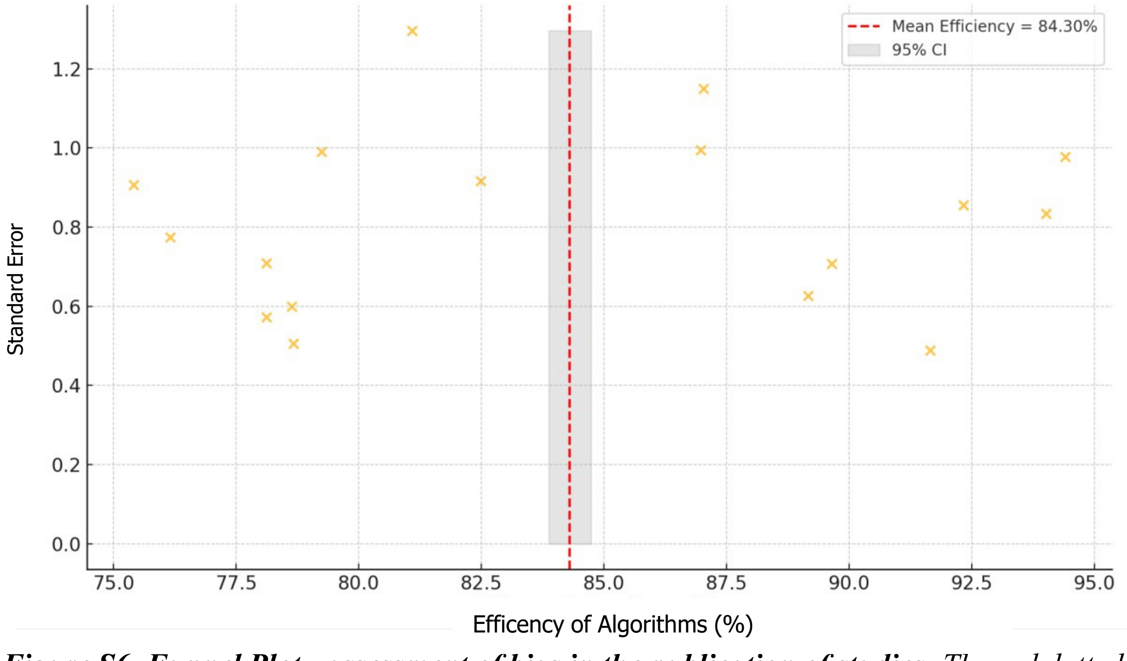

***Figure S6. Funnel Plot - assessment of bias in the publication of studies**: The red dotted line indicates the average efficiency of the algorithms. The gray shaded area represents the 95% confidence interval (CI). A symmetrical distribution of points around the red line indicates a low probability of publication bias.*

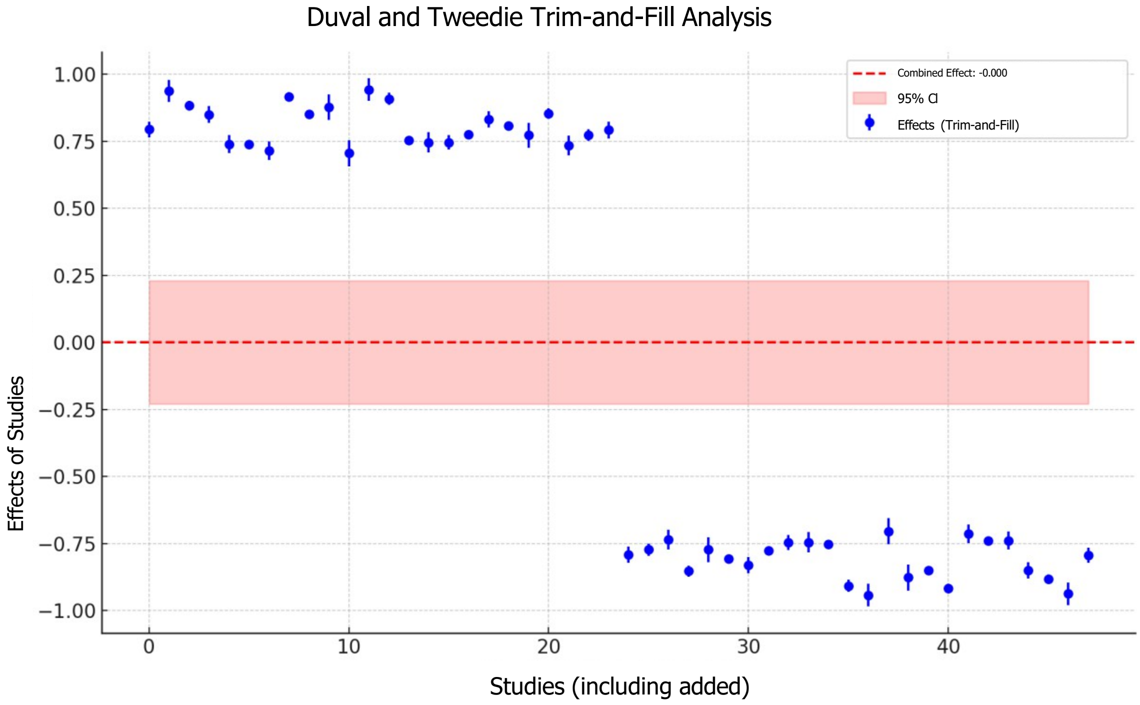

Figure S7. Results of Duval and Tweedie Trim-and-Fill Analysis. *Blue dots: Study effects, including simulated "missing" studies. Red dashed line: Combined study effect after correction. Red shading: 95% confidence interval (CI) for the combined effect. Combined Effect (Trim-and-Fill): -0.000 (practically 0, which indicates a balance of effects). 95% CI: Lower limit: -0.230; Upper limit: 0.230*

TEXT S4: RECOMMENDATIONS FOR OPTIMIZATION OF ALGORITHMS FOR INTEGRATION OF FMRI AND EEG IN STEREOTACTIC NEUROSURGERY

The results highlight the need for standardization, clinical validation, and improvements in computational efficiency. Key recommendations include:

1. *Leveraging GPU/TPU acceleration for real-time model implementation.*
2. *Establishing uniform preprocessing pipelines to reduce heterogeneity.*
3. *Conducting large clinical trials to confirm the generalizability of the model (Tables S 4 – 7).*

Table S4. Highly relevant algorithms - challenges and recommendations for optimization

Algorithm	Challenges	Optimization Recommendations
Dynamic Connectivity Models (DCM)	High model complexity, longer processing time.	Introduce adaptive models for better patient-specific adjustments. Utilize parallel data processing to reduce processing time. Integrate with real-time software platforms for intraoperative use.
Canonical Correlation Analysis (CCA)	Limited ability to handle nonlinear relationships.	Combine with kernel methods (KCCA) for nonlinear data. Integrate with deep learning models to improve signal interpretation. Develop automatic parameter tuning for better results.
Independent Component Analysis (ICA) / Joint ICA (jICA)	Sensitivity to noise and artifacts.	Implement robust ICA variants to eliminate noise. Combine with Bayesian models to improve signal identification. Optimize for real-time analysis by reducing computational demands.
Multimodal Fusion	High computational complexity, need for data synchronization.	Develop automated pipelines for EEG, fMRI, and DTI data fusion. Use cloud-based processing for large datasets. Enhance synchronization of time series from different sources.

Table S5. Partially relevant algorithms

Algorithm	Challenges	Optimization Recommendations
Neural Transformer Models	High model complexity and resource-intensive training.	Develop optimized architectures (e.g., Lightweight Transformers). Implement model quantization to reduce memory requirements. Incorporate transfer learning with pre-trained models.
Generative Adversarial Networks (GANs)	Stability issues during training and long processing time.	Switch to Wasserstein GANs (WGAN) for more stable training. Apply regularization and noise control techniques to improve results. Introduce Lightweight GAN versions for faster processing.
Deep Learning Models	Requirement for large datasets, prolonged training phase.	Use data augmentation to increase diversity of the training set. Apply model distillation for reduced complexity and faster processing. Integrate unsupervised learning for better model generalization.

Table S6. Low relevance algorithms

Algorithm	***Challenges***	***Optimization Recommendations***
Signal Decoding Models	***Limited applicability in surgical contexts.***	***Combine with neural networks for better pattern recognition. Develop task-specific decoders for surgical interventions.***
Temporal Analysis Models	***Insufficient precision for mapping functional regions***	***Implement LSTM and GRU networks for sequential analysis. Combine with Dynamic Bayesian Networks for temporal change prediction.***
Graph Theory Models	***Weak direct applicability in surgical procedures.***	***Combine with DCM models for improved functional network analysis. Develop personalized network models based on patient-specific data.***

Table S7. A detailed implementation plan for algorithm optimization in clinical practice

Algorithm Group	Specific Algorithms	Implementation Steps	Required Resources	Expected Outcomes
Highly Relevant Algorithms	DCM, CCA, ICA, jICA	Standardize input data (high-quality EEG/fMRI data). Develop adaptive models	High-quality EEG/fMRI datasets, GPU/TPU infrastructure	Faster and more precise brain mapping, safer and more successful stereotactic neurosurgery
Highly Relevant Algorithms	Multimodal Fusion, Bayesian Models	Automate pipelines for data fusion. Use cloud infrastructure for integration	Cloud servers with scalable memory. Integration with software infrastructure	More efficient mapping of functional regions through better data integration
Highly Relevant Algorithms	Real-time Feedback Systems	Develop interfaces for neurosurgical navigation systems. Test on simulation platforms	Neurosurgical software and hardware. Simulation platform for testing	Improved accuracy and reduced risks during surgeries with real-time feedback
Partially Relevant Algorithm	Neural Transformer Models, GANs, Deep Learning Models	Optimize architectures (Lightweight models). Quantize models to reduce memory usage	Computing infrastructure for deep learning. Optimized AI software	Higher precision and stability of models with reduced processing time
Partially Relevant Algorithms	Hybrid Statistical Models, Dynamic Network Models	Combine with advanced models (DCM, AI). Optimize for personalized applications	Software tools for model combinations. Support for personalized solutions	Enhanced flexibility and accuracy through model combinations and personalized applications
Low Relevant Algorithms	Signal Decoding Models, Temporal Analysis Models, Graph Theory Models	Development of specific decoders for neurosurgery. Implementation of LSTM/GRU for temporal analysis	Neurological datasets for decoder training. Advanced AI tools for temporal analysis	Greater efficiency in signal decoding and better support for clinical decisions
